# Research mapping of cannabinoids and endocannabinoid system in cancer over the past three decades: insights from bibliometric analysis

**DOI:** 10.3389/fphar.2025.1540619

**Published:** 2025-04-02

**Authors:** Yaqian Tan, Hui Xia, Qi Song

**Affiliations:** ^1^ Department of Pharmacy, The Affiliated Brain Hospital, Guangzhou Medical University, Guangzhou, China; ^2^ Key Laboratory of Neurogenetics and Channelopathies of Guangdong Province and the Ministry of Education of China, Guangzhou Medical University, Guangzhou, China; ^3^ Department of Pharmacy, Guangzhou Institute of Cancer Research, The Affiliated Cancer Hospital, Guangzhou Medical University, Guangzhou, China

**Keywords:** endocannabinoid, cannabinoids, cancer, tumor, bibliometric analysis, visualization

## Abstract

**Background:**

The cannabinoids and endocannabinoid system are thought to play critical roles in multiple signaling pathways in organisms, and extensive evidence from preclinical studies indicated that cannabinoids and endocannabinoids displayed anticancer potential. This study aimed to summarize the research of cannabinoids and endocannabinoid system in cancer through bibliometric analysis.

**Methods:**

Relevant literature in the field of cannabinoids and endocannabinoid system in cancer published during 1995–2024 were collected from the Web of Science Core Collection database. VOSviewer and SCImago Graphica were applied to perform bibliometric analysis of countries, institutions, authors, journals, documents, and keywords.

**Results:**

A total of 3,052 publications were identified, and the global output exhibited a generally upward trend over the past 3 decades. The USA had the greatest number of publications and citations in this research field. Italian National Research Council led in terms of publication, while Complutense University of Madrid had the highest total citations. Vincenzo Di Marzo was the leading author in this field with the greatest number of publications and citations. The co-occurrence of keywords revealed that the research frontiers mainly included “cannabinoids”, “endocannabinoid system”, “cancer”, “anandamide”, “cannabidiol”, “cannabinoid receptor”, “apoptosis”, and “proliferation”.

**Conclusion:**

Our results revealed that the research of cannabinoids and endocannabinoid system in cancer would receive continuous attention. The USA and Italy have made remarkable contributions to this field, supported by their influential institutions and prolific scholars. The research emphasis has evolved from basic functional characterization to mechanistic exploration of disease pathways and translational applications within multidisciplinary framework.

## 1 Introduction

Cannabinoids, also known as phytocannabinoids, are compounds isolated from the marijuana plant (Ramer et al., 2021). Cannabinoids have been applied clinically to treat various cancer-related symptoms, such as nausea, vomiting, and cancer pain, and has brought benefits to cancer patients which improved their quality of life (Tramèr et al., 2001; Khasabova et al., 2012). The endocannabinoid system is a vital mechanism controlling multiple cellular growth and developmental processes in organisms (Moreno et al., 2019). Dysregulation of this system may result in abnormal proliferation, vascularization, and tumor invasion, leading to the development of various cancers (Cherkasova et al., 2022). The endocannabinoid system contains cannabinoid receptors, endocannabinoids, and the enzymes that produce and degrade endocannabinoids (Fonseca et al., 2018). Accumulating evidence from preclinical research have indicated the anticancer properties of the cannabinoids and endocannabinoid system in various types of cancer (Velasco et al., 2012; Hermanson and Marnett, 2011; Wang et al., 2018), whereas other evidence suggested a cancer-promoting effect (Preet et al., 2011; McAllister et al., 2021) as well as interactions with standard antitumor treatments (Jiang et al., 2013; Taha et al., 2019; Bar-Sela et al., 2020).

Over the past few decades, the research of cannabinoids and endocannabinoid system in anticancer therapies have been summarized in several reviews (Ramer et al., 2021; Moreno et al., 2019; Cherkasova et al., 2022; Hermanson and Marnett, 2011; Irrera et al., 2021; Ramer and Hinz, 2016; Hinz and Ramer, 2022). However, previous literature primarily concentrated on the mechanisms of action and clinical applications, and lacked comprehensive coverage of this theme in a larger scope. The approach of bibliometrics provides quantitative insights of a specific research area through mathematical and statistical methods, and has been widely applied in literature analysis (Chen, 2004; Chen and Song, 2019). In this context, we therefore conducted a bibliometric analysis to delve into the landscape and hotspots of the research on cannabinoids and endocannabinoid system in cancer. We believe our results of the research status, collaborative networks, and key research directions, would provide theoretical reference for future exploration of this area.

## 2 Materials and methods

**FIGURE 1:**
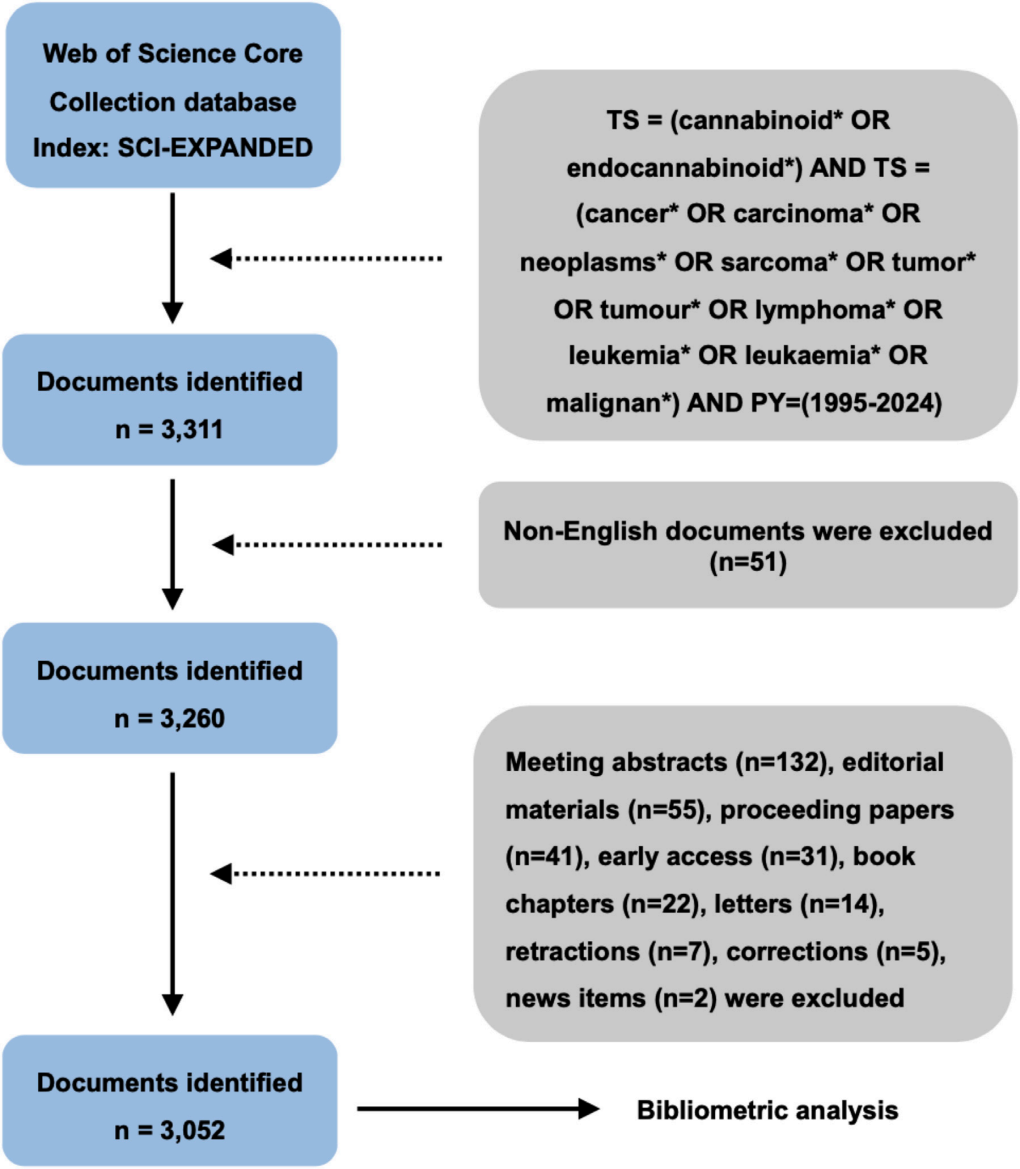
Flow diagram of literature screening process.

The literature data were extracted from the Science Citation Index Expanded (SCIE) of Web of Science Core Collection (WoSCC) database. Although WoSCC is not the largest literature database, it is most widely used in bibliometric studies due to its advantages in subject categorization, citation information coverage, and journal quality (Wang and Waltman, 2016; Birkle et al., 2020; Adriaanse and Rensleigh, 2013; Kokol and Vošner, 2018; Pranckutė, 2021; Mongeon and Paul-Hus, 2016). In this study, the time period of publications was ranged from 1 January 1995 to 31 October 2024. The search term was set as follows: TS = (cannabinoid* OR endocannabinoid*) AND TS = (cancer* OR carcinoma* OR neoplasms* OR sarcoma* OR tumor* OR tumour* OR malignant* OR leukaemia* OR leukemia* OR lymphoma*). We conducted the data acquisition on a single day (31 October 2024) to eliminate potential bias from daily database updates. Given the dominance of English in scholarly communication and the fact that 98% of SCIE publications are written in English (Ramírez-Castañeda, 2020; Hamel, 2007), we restricted the publication language to English to avoid cross-linguistic ambiguity. In addition, original articles and reviews are regarded as the primary sources of bibliometric analysis considering their high coverage and complete citation information (Kalantari et al., 2017; Tsay and Shu, 2011). Therefore, document types including meeting abstracts, editorial materials, proceeding papers, early access, book chapters, letters, retractions, corrections, and news items, were excluded from the bibliometric analysis (Figure 1).

Data were exported to plain text file format, and data mining and visualization were conducted using VOSviewer (version 1.6.18) and SCImago Graphica (version 1.0.45). VOSviewer is a commonly used tool under JAVA environment that offers the functions of massive bibliometric data processing and mapping (van Eck and Waltman, 2010; Arruda et al., 2022; van Eck and Waltman, 2017). In this study, VOSviewer was employed to analyze and visualize the collaborative relationships and thematic clusters in several dimensions, including countries, institutions, authors, journals, documents, and keywords. The visualization of country co-authorship and keyword co-occurrence were performed using SCImago Graphica. The counting method of co-authorship and co-occurrence was set as “Full counting”, and the unit of keywords analysis was “All keywords”. Prior to data analysis, we performed data standardization to eliminate ambiguation of author names and institution names. In addition, due to the default setting of VOSviewer, some country/region data required integration. For instance, the terms of “England”, “Scotland”, “North Ireland”, and “Wales” were integrated into a single term, “UK”.

## 3 Results

### 3.1 Overview of global output

**FIGURE 2:**
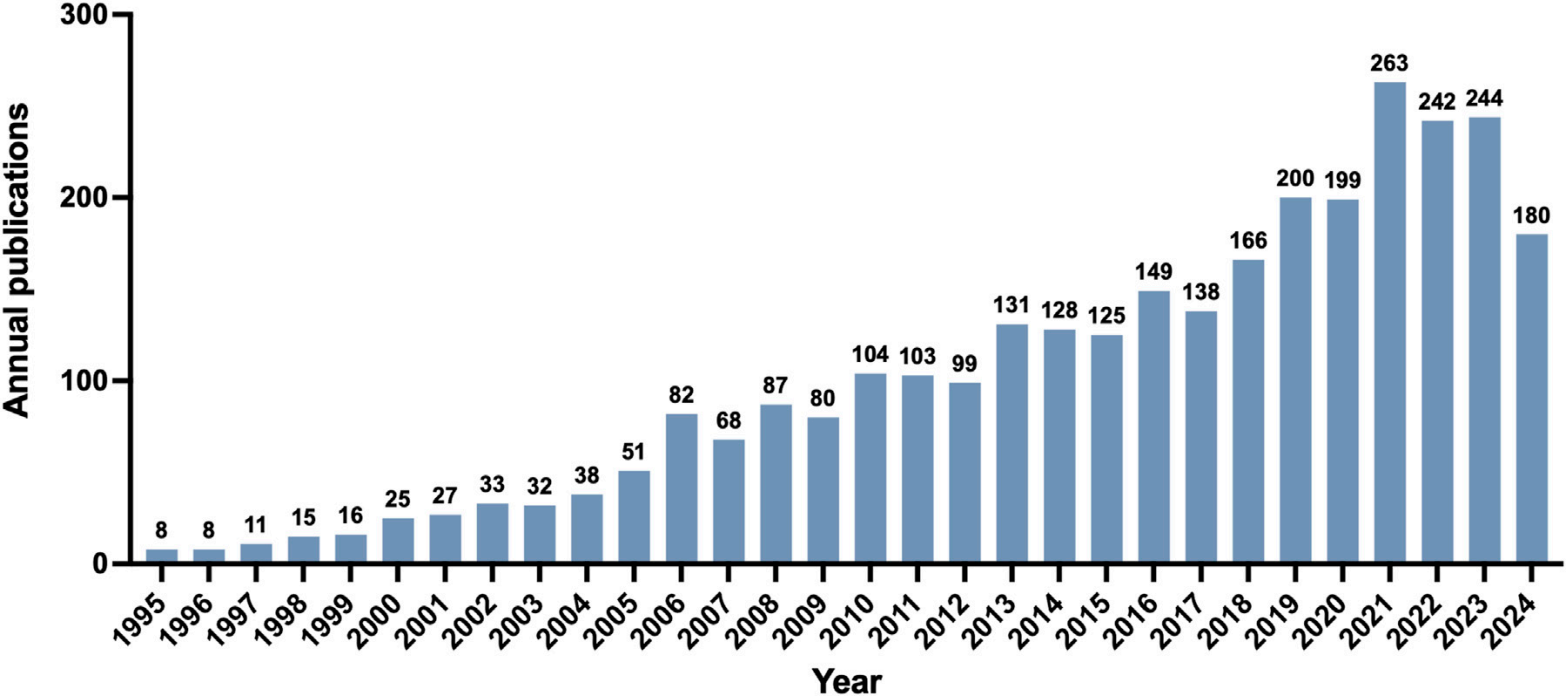
Global trend of annual publications from 1995 to 2024.

A total of 3,052 publications were identified after data screening. As demonstrated in Figure 2, the annual global output from 1995 to 2024 reflected a fluctuating upward trend, indicating the increasing attention of this field over the past 3 decades. The number of annual global output was below 100 during 1995–2009. The number of publications began to exceed 100 in 2010 (n = 104), and subsequently peaked in 2021 with 263 publications.

### 3.2 Contributions of countries, institutions, and authors

**FIGURE 3:**
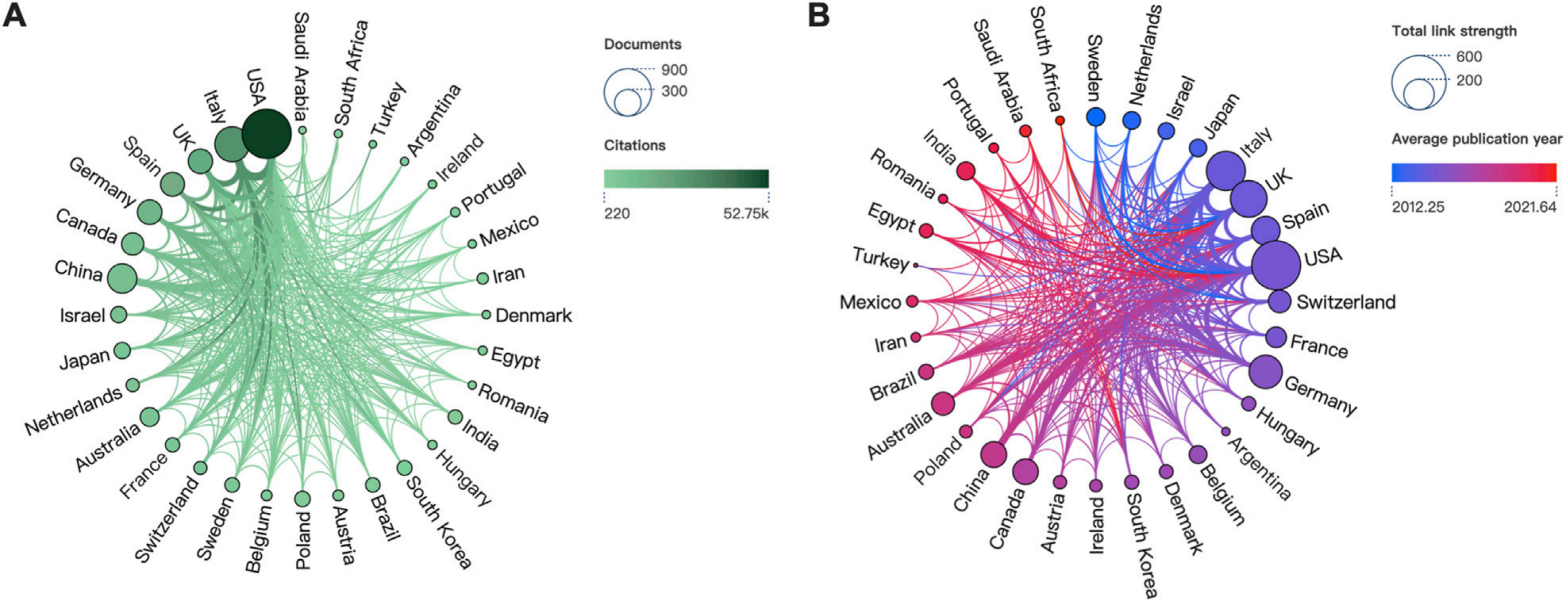
Visualization networks of countries. **(A)** Contributions and citations of countries. **(B)** Historiograph and cooperation between countries.

A total of 86 countries have published studies related to cannabinoids and endocannabinoids in cancer. Figure 3 depicted the overview of countries on this research theme. In Figure 3A, the size of the node reflected the scientific outputs of countries, while the color represented their total citations. The top three productive countries were the USA (n = 892), Italy (n = 447), and China (n = 319), whereas the top three most-cited countries were the USA (n = 52,754), Italy (n = 27,837), and the UK (n = 16,979). The visualization of country collaboration networks and academic activities were illustrated in Figure 3B. The node size indicated the link strength between countries, whereas the color represented their academic activity. We found the thickest link was between the USA and Italy, reflecting their close collaborations. Concerning academic activity, Sweden exhibited an earlier contribution in this field, whereas South Africa displayed a more recent activity.

A total of 3,362 institutions participated in this research domain, with Italian National Research Council (n = 93), Complutense University of Madrid (n = 87), and University of Naples Federico II (n = 61) ranked as the leading three most productive institutions (Table 1). As summarized in Table 2, Complutense University of Madrid (n = 9,983) was the institution with the highest citations, followed by Italian National Research Council (n = 9,418) and University of Naples Federico II (n = 5,329).

**TABLE 1:** The top ten most productive institutions.

Rank	Institution	Publications
1	Italian National Research Council	93
2	Complutense University of Madrid	87
3	University of Naples Federico II	61
4	The Hebrew University of Jerusalem	54
5	University of Salerno	54
6	Umeå University	39
7	University of Minnesota	34
8	University of Teramo	34
9	Indiana University	33
10	University of Pisa	33

**TABLE 2:** The top ten most-cited institutions.

Rank	Institution	Total citations
1	Complutense University of Madrid	9,983
2	Italian National Research Council	9,418
3	University of Naples Federico II	5,329
4	The Hebrew University of Jerusalem	4,721
5	National Institute on Alcohol Abuse and Alcoholism	4,613
6	University of Salerno	3,847
7	Spanish National Research Council	2,808
8	University of Aberdeen	2,524
9	University of Rome Tor Vergata	2,452
10	Indiana University	2,433

**FIGURE 4:**
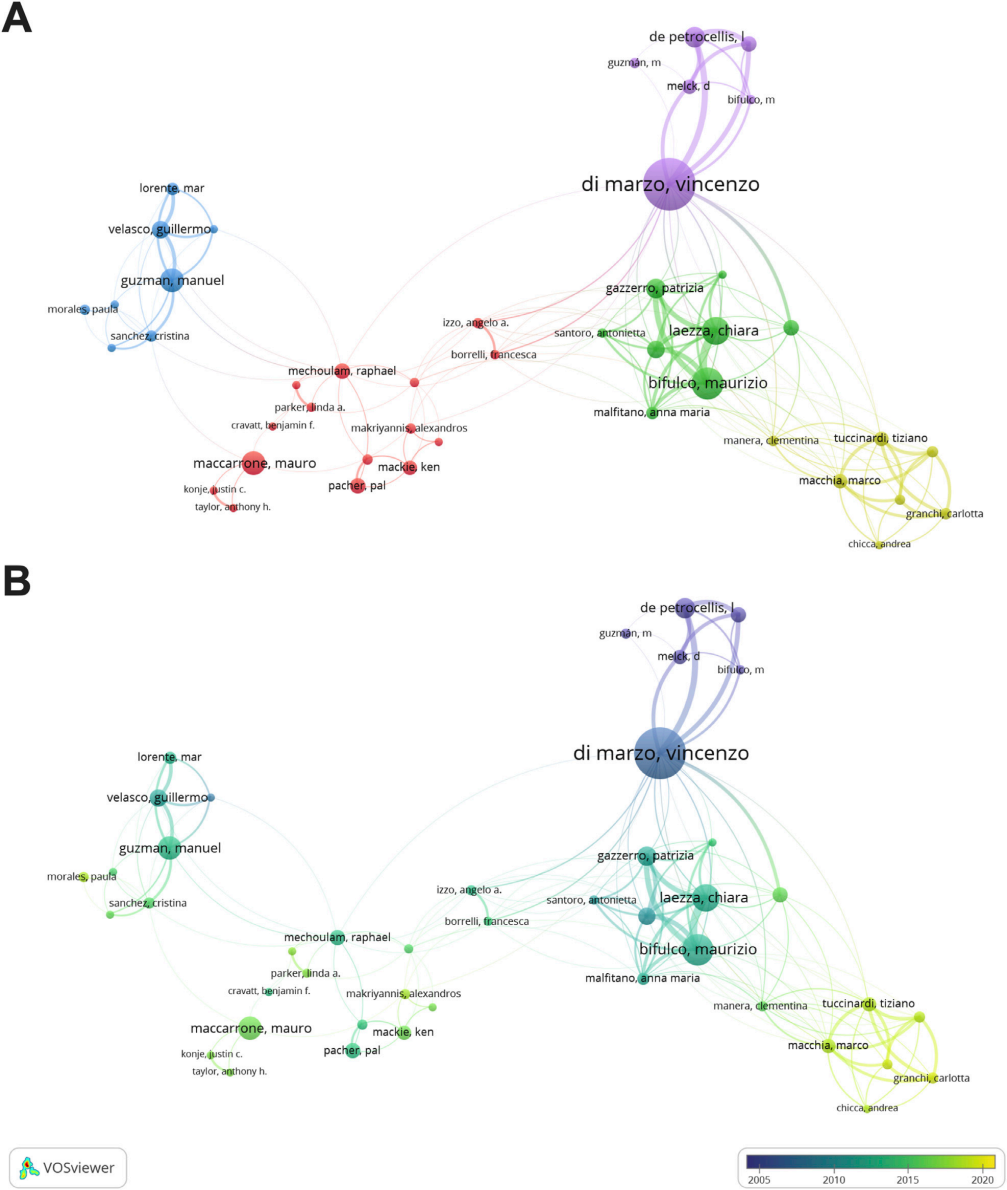
Visualization networks of authors. **(A)** Contributions and cooperation between authors. **(B)** Historiograph of author co-authorship.

In this study, we found 14,087 authors contributed to the research of cannabinoids and endocannabinoid system in cancer. Figure 4 depicted the co-authorship visualization atlas. The outputs of authors were indicated by the size of the nodes, while the connecting lines between nodes denoted collaboration strength among authors. The color of nodes indicated distinct collaboration clusters (Figure 4A) or the academic activities of authors (Figure 4B). Vincenzo Di Marzo was one of the most fruitful scholars in this field, ranking first in publication (n = 61) and total citation (n = 4,364). Moreover, Vincenzo Di Marzo, Maurizio Bifulco, Mauro Maccarrone, Manuel Guzman, and Marco Macchia were the leading scholars in this domain and formed close collaborations.

### 3.3 Productive journals and top-cited documents

In total, we detected 1,023 journals published literature in this theme. Table 3 listed the leading journals in publications. The most fruitful journal in this research area was *International Journal of Molecular Sciences* (n = 119), followed by *British Journal of Pharmacology* (n = 60) and *Molecules* (n = 59). There were eight of the top 10 most productive journals located in Q1 region according to the Journal Citation Reports 2023, and *British Journal of Pharmacology* had the highest impact factor (IF) of 6.8. As shown in Table 4, *British Journal of Pharmacology* (n = 6,690), *International Journal of Radiation Oncology Journal of Biological Chemistry* (n = 3,550), and *European Journal of Pharmacology* (n = 2,471) were the top three journals in citations. Moreover, seven of the top 10 most-cited journals were distributed in Q1 region, and *Cancer Research* had the highest IF of 12.5. Together, these results of the journal analysis indicated their high relevance and quality in the field. Table 5 presented the top 10 most-cited documents in this field. These documents were published between 2003 and 2022, and they were all cited more than 500 times. The review article titled “The endocannabinoid system as an emerging target of pharmacotherapy” published in *Pharmacological Reviews* by Pál Pacher et al. had the highest citation of 1,570.

**TABLE 3:** The top ten most fruitful journals.

Rank	Journal	Publications	IF 2023	JCR region
1	*International Journal of Molecular Sciences*	119	5.6	Q1
2	*British Journal of Pharmacology*	60	6.8	Q1
3	*Molecules*	59	4.2	Q2
4	*Cancers*	52	4.5	Q1
5	*European Journal of Pharmacology*	52	4.2	Q1
6	*Cannabis and Cannabinoid Research*	42	3.1	Q2
7	*Frontiers in Pharmacology*	37	4.4	Q1
8	*PLoS One*	36	2.9	Q1
9	*Life Sciences*	33	5.2	Q1
10	*Scientific Reports*	31	3.8	Q1

**TABLE 4:** The top ten most-cited journals.

Rank	Journal	Total citations	IF 2023	JCR region
1	*British Journal of Pharmacology*	6,690	6.8	Q1
2	*Journal of Biological Chemistry*	3,550	4	Q2
3	*European Journal of Pharmacology*	2,471	4.2	Q1
4	*International Journal of Molecular Sciences*	2,326	4.9	Q1
5	*Cancer Research*	2,229	12.5	Q1
6	*Journal of Neuropathology and Experimental Neurology*	2,016	3.2	Q2
7	*Journal of Pharmacology and Experimental Therapeutics*	1,898	3.1	Q1
8	*FASEB Journal*	1,585	4.4	Q1
9	*Prostaglandins Leukotrienes and Essential Fatty Acids*	1,548	2.9	Q3
10	*Molecular Cancer Therapeutics*	1,486	5.3	Q1

**TABLE 5:** The top ten most-cited documents.

Rank	First author	Year	DOI	Citation
1	Pál Pacher	2006	10.1124/pr.58.3.2	1,570
2	Angelo A Izzo	2009	10.1111/j.1476-5381.2010.01166.x	997
3	Ethan B Russo	2011	10.1001/jama. 2015.6358	947
4	Luciano De Petrocellis	2011	10.1136/gutjnl-2021-326789	658
5	Dina G Tiniakos	2010	10.1523/JNEUROSCI.4540-04.2005	655
6	Belén G Ramírez	2005	10.1016/j.tips. 2009.07.006	620
7	Takaaki Higashi	2017	10.2165/00003088-200342040-00003	606
8	Willem M de Vos	2022	10.1111/j.1476-5381.2011.01238.x	559
9	Franjo Grotenhermen	2003	10.1146/annurev-pathol-121808-102132	531
10	Penny F Whiting	2015	10.1016/j.addr. 2017.05.007	502

### 3.4 Keywords co-occurrence analysis

**FIGURE 5:**
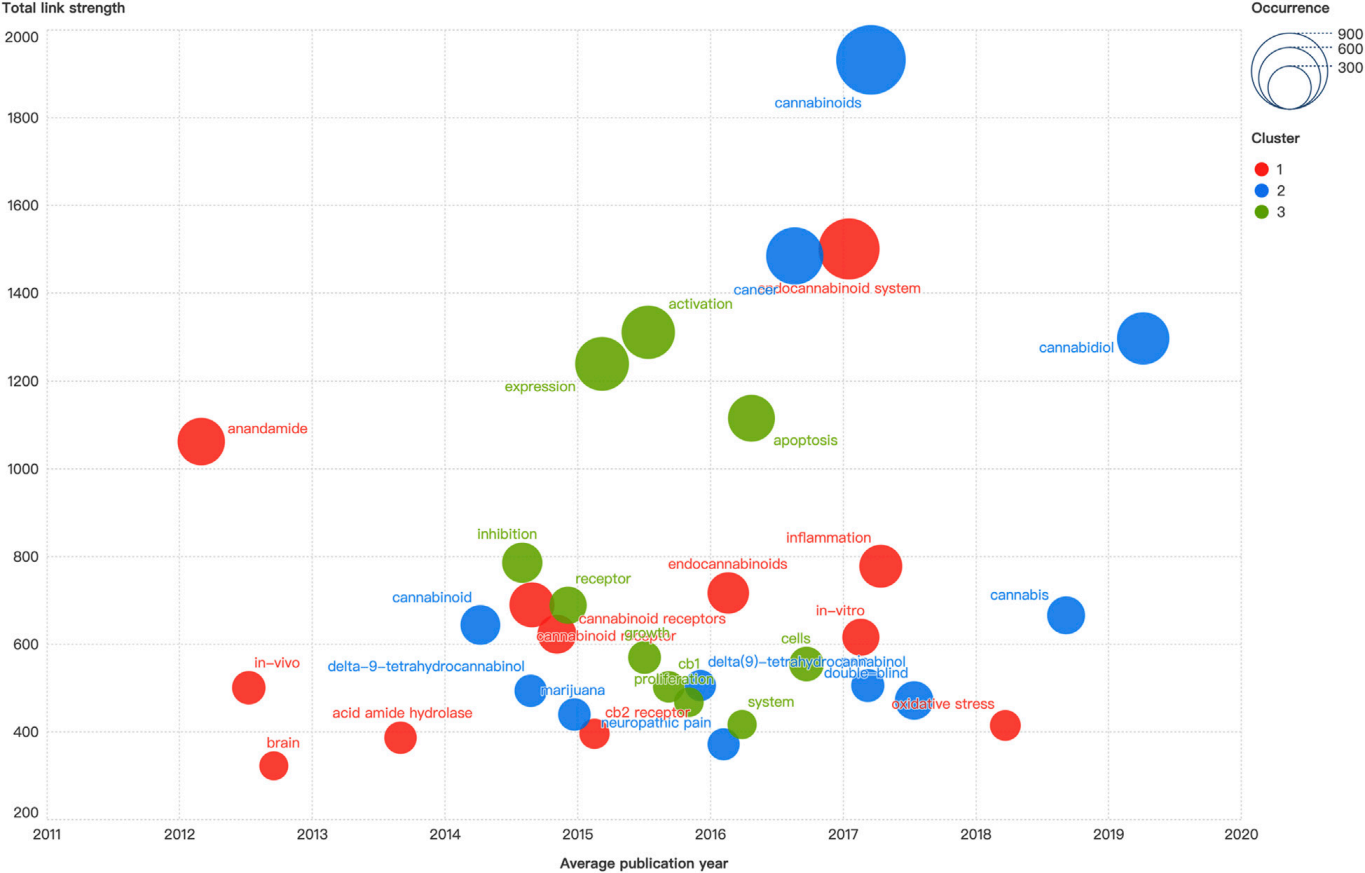
The distribution and historiograph of keywords co-occurrence.

A total of 11,137 relevant keywords were collected, and the top 30 keywords with the strongest occurrence were demonstrated in Figure 5. The minimum threshold of keywords was adjusted to 120 to ensure readability of the graph. The keyword “cannabinoids” had the highest occurrence and the strongest link strength. The keyword with the earliest appearance was “anandamide”, and the newly emerged keyword was “cannabidiol”. The identified keywords were clustered into three distinct groups labeled by different colors. The red cluster encompassed keywords such as “endocannabinoid system”, “anandamide”, “cannabinoid receptor”, “endocannabinoids”, “inflammation”, “oxidative stress”, “cb2 receptor”, and “acid amide hydrolase”. The blue cluster included keywords of “cannabinoids”, “cannabidiol”, “cancer”, “cannabis”, “marijuana”, “neuropathic pain”, and “delta-9-tetrahydrocannabinol”. In the green cluster, “activation”, “expression”, “apoptosis”, “inhibition”, “receptor”, “cb1”, and “proliferation” were the main nodes included. The average publication year of the keywords demonstrated that the research frontiers were shifted from basic functional characterization of cannabinoid and endocannabinoid (e.g., “anandamide” and “acid amide hydrolase”) as well as cannabinoid receptors (e.g., “cb1” and “cb2 receptor”), to mechanisms of diseases (e.g., “neuropathic pain”, “proliferation”, “apoptosis”, and “oxidative stress”), and ultimately the translational applications within multidisciplinary framework of molecular biology, pathophysiology, pharmacology, immunology, and oncology.

## 4 Discussion

### 4.1 General trends and structures

In this study, we conducted a comprehensive bibliometric analysis in research concerning cannabinoids, endocannabinoid system, and cancer. The increasing tendency in global output reflected the growing recognition of this field, which was likely driven by the rapid developments in disciplines of physiology, pharmacology, biochemistry, and oncology. However, in recent years, the advances in anticancer therapies, including immune checkpoint inhibitors (ICIs), antibody-drug conjugates, and chimeric antigen receptor T cells, have attracted a substantial amount of attention and resources. In contrast, the limited progress in new target and unelucidated clinical efficacy of cannabinoids might have led to the recent slowdown in the development of this field (Pramesh et al., 2022; Skórzewska and Gęca, 2024; Chen et al., 2022).

As for productive countries, although the USA led in national contributions, Italy was more prominently in institutional output, possessing half of the top 10 institutions. A more in-depth analysis revealed that the majority of the top 100 institutions (n = 33) were still located in the USA, exhibiting a scattered and evenly distributed institutional output. This pattern might be attributed to differences among states in funding sources, research scale, and cannabis-related legal restrictions (Dickson et al., 2019; Cooper et al., 2021). Meanwhile, due to cultural acceptance and regulatory differences, cannabis research was more prevalent in European countries, such as Italy and Spain (Krcevski-Skvarc et al., 2018; Ransing et al., 2022). As the core of scientific research system in Italy, the Italian National Research Council plays a fundamental role in coordinating research activities among institutions and has contributed significantly to the research output (Tuzi, 2005). On the other hand, although Spain ranked sixth in the number of publications (n = 212), the Complutense University of Madrid held second in institutional output (n = 87), contributing 41.0% of the national production. The fruitful researchers, including Manuel Guzman, Guillermo Velasco, Mar Lorente, Cristina Sánchez, and Maria Salazar Roa, have formed close collaborations and contributed significantly to the academic status of this institution (Velasco et al., 2012; Maccarrone et al., 2014; Guzmán, 2003; Velasco et al., 2016a).

From the perspective of author contribution, the research groups led by Vincenzo Di Marzo, Maurizio Bifulco, Mauro Maccarrone, Manuel Guzman, and Marco Macchia, excelled in this domain and greatly advanced its development. Vincenzo Di Marzo pioneered in this topic and provided profound insights into the metabolic pathways, physiological functions, and anticancer actions, of endocannabinoids (Di Marzo, 2008; Marzo et al., 2004). Maurizio Bifulco had close connection with Vincenzo Di Marzo and primarily investigated the endocannabinoid system, cannabinoid receptor, cannabinoid, and their roles in cancer (Ligresti et al., 2003; Ligresti et al., 2006a; Pisanti et al., 2017). Mauro Maccarrone was a key scholar in cannabinoid research, and focused on endocannabinoid signaling, lipoxygenase pathway, and neuroinflammation (Maccarrone et al., 2000; Maccarrone et al., 2015). Manuel Guzman made fruitful achievements in the mechanisms of cannabinoid and cannabinoid receptors in oncology and neurology, and collaborated closely with Mauro Maccarrone (Velasco et al., 2012; Maccarrone et al., 2014; Guzmán, 2003). Marco Macchia was active more recently in this field, and concentrated on cannabinoid receptor-targeting pharmacological strategies in cancer treatment (Manera et al., 2015; Bertini et al., 2016).

Literature on this topic tended to be published in specialized journals of molecular sciences, pharmacology, biochemistry, and oncology. Moreover, the majority of journals were Q1 journals, and ranged 2.9 to 12.5 in IF. These findings indicated the high quality of the literature, and would be helpful for researchers seeking the core journals in the field. Besides, the most-cited publication was the review by Pál Pacher et al. titled “The endocannabinoid system as an emerging target of pharmacotherapy”. This review comprehensively summarized the knowledge status of the endocannabinoid system as a target of pharmacotherapy (Pacher et al., 2006).

### 4.2 Research development pattern

Publications on anticancer effects of cannabinoids and endocannabinoid system have accumulated over the last few decades. In the early 1970s, by using Lewis lung adenocarcinoma mice model, Munson et al. first discovered that orally administration of tetrahydrocannabinol inhibited tumor growth, suggesting the antiproliferative effects of cannabinoids (Munson et al., 1975). In the 1980s, Howlett et al. systematically introduced the cannabinoid receptors in their identification, distribution, and pharmacological properties (Howlett et al., 2002). The research of cannabinoid regulation of cell growth signaling was advanced after the characterization of cannabinoid receptors. From the 1990s, numerous findings from preclinical animal studies and human clinical work revealed the antitumor effects of endogenous cannabinoids and synthetic cannabinoids among various cancers, including lymphoma, pancreatic cancer, glioma, breast cancer, and prostate cancer (Carracedo et al., 2006a; Blázquez et al., 2006; Ligresti et al., 2006b). Follow-up studies from the 2000s focused on the mechanisms of cannabinoid-induced apoptosis and growth inhibition (Galve-Roperh et al., 2000a). Nowadays, the interest in cannabinoids and endocannabinoid system have increased due to their effects of antibacterial, anti-inflammatory, anti-anxiety, and neuroprotective (Luz-Veiga et al., 2023; Aljobaily et al., 2022). However, considering the psychoactivity and side effects of cannabinoids (Irrera et al., 2021; Abrams, 2016), their pharmacological potential is yet to be fully realized.

The endocannabinoid system is a vital mechanism controlling multiple cellular growth and developmental processes in organisms (Moreno et al., 2019). Dysregulation of this system may result in abnormal proliferation, vascularization, and tumor invasion (Cherkasova et al., 2022). The endocannabinoid system consists of G protein-coupled cannabinoid receptors, endocannabinoids, and enzymes that produce and degrade endocannabinoids (Fonseca et al., 2018). On the other hand, cannabinoids are compounds derived from the marijuana plant, and showed inhibitory effects of tumor proliferation, invasion, metastasis, and angiogenesis (Ramer et al., 2021; Velasco et al., 2012). Delta-9-tetrahydrocannabinol is the major component of phytocannabinoid, responsible for the psychoactive effects of marijuana (Maia et al., 2023). Previous reviews have summarized the evidence from animal experiments, clinical studies, and epidemiological research, indicating that non-psychoactive cannabinoids, such as cannabidiol, has potential therapeutic effects of anti-inflammatory, analgesic, and anxiolytic (Pertwee, 2006; Blessing et al., 2015). Besides phytocannabinoids, endocannabinoids also have the ability to activate cannabinoid receptors (Fonseca et al., 2013). The two well-known endocannabinoids are anandamide and 2-arachidonoylglycerol (Lim et al., 2023), and they are closely related to the regulation of pain, emotion, and immunity (Popescu-Spineni et al., 2022).

Effects of cannabinoids are thought to be mediated via 2 G protein-coupled receptors, namely, cannabinoid receptors type 1 (CB1) and cannabinoid receptors type 2 (CB2) (Howlett, 1995). The former is primarily expressed in central nervous system, whereas the latter is mostly found in the peripheral nervous system and immune cells (Devane et al., 1992). Moreover, an earlier *in vitro* study has reported the antitumor effect of cannabinoids via transient receptor potential vanilloid type 1 (TRPV1), reflecting the attractive research prospect of selective targeting TPRV1 (Fonseca et al., 2018). Aside from the direct activation of cannabinoid receptors, another approach is to increase endocannabinoid levels by inhibiting endocannabinoid-degrading enzymes (Schwarz et al., 2018). Therefore, we envision that selective inhibitors of endocannabinoid degrading enzyme would have promising research potential.

Numerous findings from rodent and *in vitro* models have suggested that cannabinoid receptors exert anticancer effects through diverse mechanisms, such as cell death stimulation, cell proliferation inhibition, and angiogenesis inhibition (Garmpis et al., 2022). Several pathology studies using surgical specimens from cancer patients have indicated that upregulated levels of CB1 or CB2 were associated with reduced survival and increased tumor metastasis and recurrence (Hashemi et al., 2020; Klein Nulent et al., 2013; Larrinaga et al., 2013; Wu et al., 2012). Other findings from clinical immunohistochemistry studies and rodent model experiments reported that higher CB1 and CB2 levels were linked to poor clinical outcomes in patients of renal cell carcinoma (Wang et al., 2018), head and neck squamous carcinoma (Klein et al., 2013), breast cancer (Blasco-Benito et al., 2019), and colorectal cancer (Martínez-Martínez et al., 2015). However, others reported the association between CB1/CB2 upregulation and longer survival time in hepatocellular carcinoma (Xu et al., 2006) and non-small cell lung cancer (Preet et al., 2011; McAllister et al., 2021). Thus, these controversial findings on the effects of cannabinoid receptors in cancer might suggest the necessity to distinguish exact cell types, such as tumor tissue, molecular tumor subtype, or immune cells, in order to explore more accurate prognostic property from the parameters. Further endeavors are required to thoroughly evaluate the potential role of cannabinoid receptors as biomarkers in cancer prevention and treatment.

### 4.3 Research hotspots

Cannabinoids have been applied clinically to treat various cancer-associated symptoms, such as nausea, vomiting, and cancer pain, which brought additional benefits for cancer patients and their quality of life (Tramèr et al., 2001; Khasabova et al., 2012). As for antitumor properties of cannabinoids, several mechanisms have been proposed, including cytostatic effects, apoptosis induction, and inhibition of neo-angiogenesis (Bifulco et al., 2006). Extensive studies using animal tumor models have revealed an association between cannabinoid-induced autophagy and apoptosis (Lorente et al., 2011). Salazar et al. first demonstrated that tetrahydrocannabinol induced autophagy in glioma cells via CB1 phosphorylation and endoplasmic reticulum stress (Salazar et al., 2009). Other preclinical work revealed that classic anticancer agents may benefit from such endoplasmic reticulum stress and autophagy inhibitors (Sui et al., 2013; Xu et al., 2014). In addition to cell death promoting effects, cannabinoids also showed antiangiogenic effects by inhibition of vascular endothelial growth factor receptors (VEGFR), VEGFR one and VEGFR2 (Casanova et al., 2003). Likewise, synthetic cannabinoids exhibited sustained inhibition of angiogenic process rather than inducing angiogenesis (Maia et al., 2023).

Besides, cannabinoids have been reported to greatly affect immune cells by altering multiple genes involved in immune response, immune cell apoptosis, and cell proliferation (Hu et al., 2020). Findings from immunodeficient mice model have shown a strong antitumor effect of the synthetic cannabinoid (Blázquez et al., 2006). The anti-inflammatory and immunosuppressive effects of cannabinoids have prompted the combination therapy with ICIs, though the clinical outcomes remained unclear. A few clinical studies strongly suggested that medicinal cannabis can improve the tolerability of cancer patients to ICIs treatment (Vigano et al., 2025), while no significant benefits on overall survival or progression free survival were noted (Bar-Sela et al., 2020; Biedny et al., 2020). On the contrary, some studies have indicated that the concurrent use of cannabinoids with ICIs can induce adverse effects on clinical outcomes (Sarsembayeva and Schicho, 2023).

The endocannabinoid system has emerged as a promising new target for pharmacological intervention in growth suppression and apoptosis induction in many cancer cells (Prather et al., 2013; Galve-Roperh et al., 2000b; Carracedo et al., 2006b; Massi et al., 2004; Tomko et al., 2019). Of note, although cannabinoids were found to display apoptotic effects in breast cancer cells (Caffarel et al., 2012), others studies reported protumorigenic effects of cannabinoids in breast cancer (McKallip et al., 2005; Zhu et al., 2000). Due to the complexity of endocannabinoid system in regulating cancer biology pathways, it may exert different effects on antiproliferative, antiangiogenic, anti-metastatic, and anti-inflammatory, which might explain its uncertainty in anticancer treatments (Mandrika et al., 2010). Interestingly, regarding lung cancer, although cannabis contains similar toxins and carcinogens to tobacco, there is currently no conclusive evidence that cannabis is associated with an increased risk of lung cancer (Jett et al., 2018; Yarlagadda et al., 2019). On the other hand, while some clinical evidence supported the benefits of cannabis in the treatment of lung cancer, it remained debatable to be included in official cancer treatment guidelines (Smith et al., 2015).

Regarding combined therapies, the combined use of cannabinoids was found to enhance the antiproliferative effects of various classical chemotherapeutic agents, including temozolomide, gemcitabine, paclitaxel, and of 5-fluorouracil (Shrivastava et al., 2011; Donadelli et al., 2011; Miyato et al., 2009; Gustafsson et al., 2009). However, the pharmacokinetic properties of cannabinoids need to be carefully considered as well. Previous findings have indicated that cannabinoids inhibited cytochrome P450 activity, which may consequently increase the toxicity and reduce the efficacy, of chemotherapeutic agents (Jiang et al., 2013; Yamaori et al., 2011). Therefore, the interaction between chemotherapeutic agents and cannabinoids would be a noteworthy issue that requires further clarification in future clinical studies. Additionally, the interaction between cannabinoids and ICIs has also gained attention. Clinical trial evidence has revealed the immunosuppressive effects of cannabinoids during concurrent nivolumab treatment (Taha et al., 2019; Bar-Sela et al., 2020). Thus, careful consideration should be given on patients treated with ICIs and cannabinoids. In targeted therapies, Velasco et al. found that cannabinoids is able to enhance the antineoplastic activity of targeted therapies by regulating protein kinases, such as anaplastic lymphoma receptor tyrosine kinase, epidermal growth factor receptor kinase, and extracellular signal-regulated kinase, demonstrating promising anticancer potential (Velasco et al., 2016b).

Interestingly, our results of keywords analysis revealed that several hotpots were more frequently occurred in recent years. First, with the legislation changes in the USA that medicinal use of cannabis has become more normalized, its potential in managing cancer-related symptoms and improving quality of life for cancer patients are becoming particularly attractive these years (McClure et al., 2023; Amin et al., 2024). Moreover, there has been growing evidence supporting the antitumor role of cannabis in an expanding list of cancers (Alsalamat et al., 2024). Although cannabidiol and tetrahydrocannabinol are the primary chemical compounds derived from cannabis, a scientific favor of cannabidiol over tetrahydrocannabinol was observed due to the multi-targeted property and less psychotropic effects of cannabidiol (Maayah et al., 2020; Mashabela and Kappo, 2024). Furthermore, evidence suggested that cannabidiol promotes apoptosis in cancer cells by targeting oxidative stress through oxidative cellular damage (Mokoena et al., 2022). Hence, cannabidiol would be a promising non-intoxicating target which is crucial for optimizing the therapeutic potential in cancer treatment.

### 4.4 Outlook

Overall, cannabinoids and endocannabinoid system have demonstrated potential therapeutic benefits for cancer. However, challenges have simultaneously emerged due to their extensive and complex influence on cancer progression (Skórzewska and Gęca, 2024). One of the main challenges lies in the step of clinical translation. Current evidence from clinical trials suggested that studies of cannabinoids and endocannabinoid system primarily focused on the management of cancer and/or treatment-related symptoms, with variable quality of evidence (Skórzewska and Gęca, 2024; Häuser et al., 2023). Therefore, it is necessary to conduct well-designed and high-quality clinical trials in the future. In addition, it would be of great value to combine preclinical mechanisms of action into clinical practice, such as optimal ratio of cannabinoid agents with chemotherapy and ICIs therapy (Vigano et al., 2025). Importantly, we should emphasize that the legislative issues with cannabis have constrained the collection of human trial data, thereby limiting our understanding of its side effects and drug interactions (Hayry, 2004).Thus, clinicians should carefully weigh the legal and ethical issues, such as informed patient consent and potential risks.

### 4.5 Limitations

This study should be interpreted within its limitations. First, although the use of single database facilitated our result management, the comprehensiveness of data might be potentially limited. Currently, data merging of multiple databases is often manual and requires significant effort. Future research utilizing newly developed open source tools would be highly beneficial for integrating the essence of multiple databases (Nikolić et al., 2024). In addition, due to the intrinsic limitation of bibliometrics that recent high-quality documents generally have low citation counts, these studies might be underappreciated. Future studies combining alternative metric method, such as early citation trends, would better capture the impact of emerging research in a timely manner (Wang et al., 2013).

## 5 Conclusion

In this study, we conducted a comprehensive bibliometric analysis on the research of cannabinoids and endocannabinoid system in cancer over the past 3 decades. Our results would provide referable guidance for the understanding of research emphasis on this topic, offering insights for clinical interventions and scientific inquiries.

## Data Availability

The data sets analyzed in this study are available upon request from the corresponding author.
