# Supplementary material for: Research mapping of cannabinoids and endocannabinoid system in cancer over the past three decades: insights from bibliometric analysis
Source: Front Pharmacol. 2025 Apr 2;16:1540619. doi: 10.3389/fphar.2025.1540619 (PMC12000044; doi:10.3389/fphar.2025.1540619)
Supplement: Supplementary file 1 [file DataSheet1.docx]

**Supplemental materials**

**
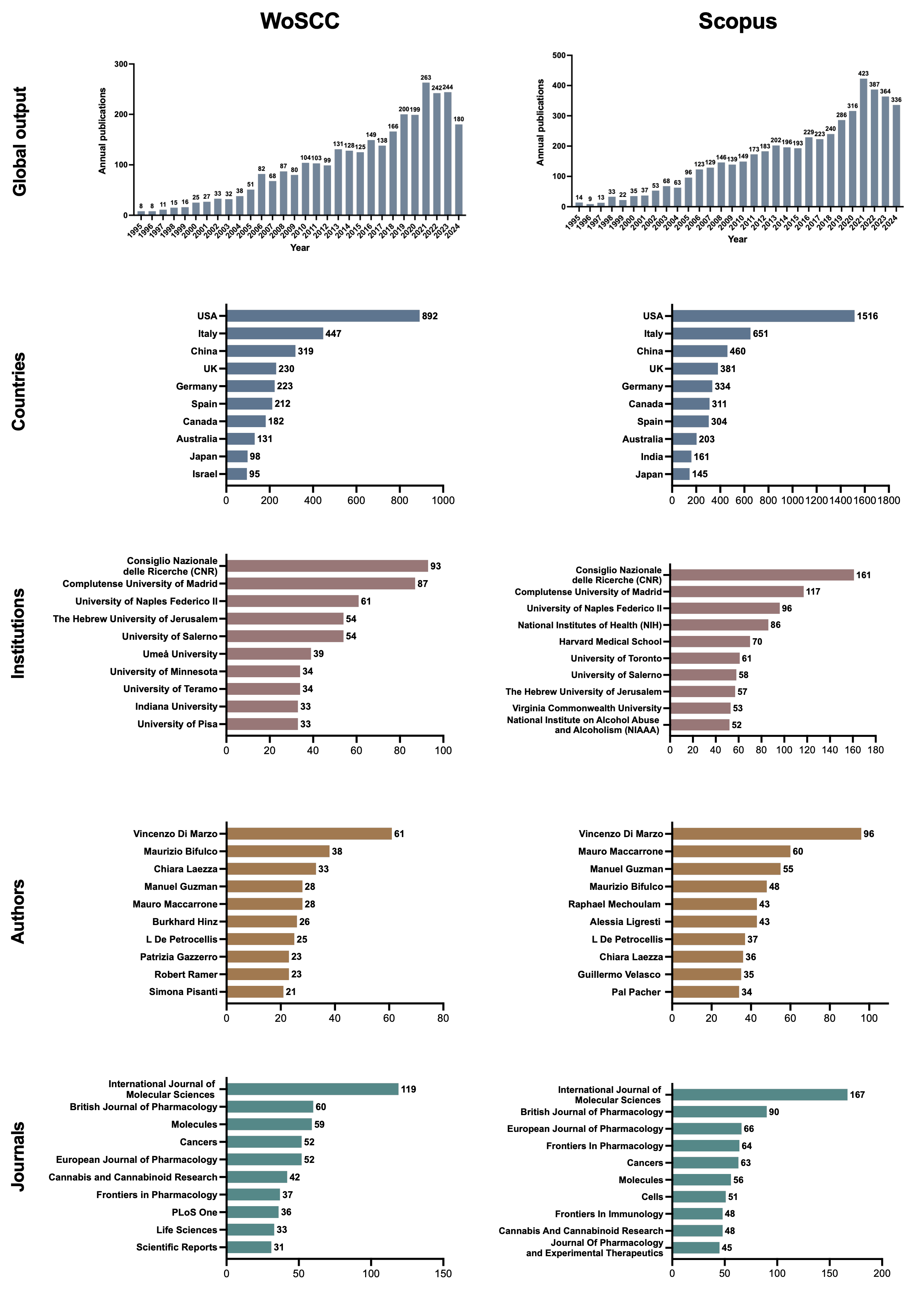
**

**Figure S1.** The annual global output and top 10 countries, institutions, authors, and journals, in WoSCC database and Scopus database.

**Table S1.** The number of collected documents and search term in WoSCC database and Scopus database.

| Database | WoSCC | Scopus |
| --- | --- | --- |
| Total number of documents | 3052 | 4880 |
| Search term | TS = (cannabinoid* OR endocannabinoid*) AND TS = (cancer* OR carcinoma* OR neoplasms* OR sarcoma* OR tumor* OR tumour* OR lymphoma* OR leukemia* OR leukaemia* OR malignan*) AND PY=(1995-2024) AND LA=(English) AND DT=(Article OR Review) | TITLE-ABS-KEY ((cannabinoid* OR endocannabinoid* ) AND ( cancer* OR carcinoma* OR neoplasms* OR sarcoma* OR tumor* OR tumour* OR malignant* OR leukaemia* OR leukemia* OR lymphoma*)) AND PUBYEAR>1994 AND PUBYEAR<2025 AND(LIMIT-TO (DOCTYPE,"ar") OR LIMIT-TO (DOCTYPE,"re")) AND (LIMIT-TO (LANGUAGE , "English")) AND (LIMIT-TO(SRCTYPE,"j")AND (LIMIT-TO(PUBSTAGE, "final"))AND(LIMIT-TO (SUBJAREA,"MEDI")OR LIMIT-TO(SUBJAREA,"BIOC")OR LIMIT-TO (SUBJAREA, "PHAR")OR LIMIT-TO(SUBJAREA,"IMMU")) |
